# Editorial: Comparative and evolutionary analyses of organelle genomes

**DOI:** 10.3389/fgene.2024.1502457

**Published:** 2024-10-22

**Authors:** Marcos A. Caraballo-Ortiz, Zhumei Ren, Xu Su, M. James C. Crabbe

**Affiliations:** ^1^ Department of Botany, National Museum of Natural History, Smithsonian Institution, Washington, DC, United States; ^2^ Department of Biology, University of Mississippi, Oxford, MS, United States; ^3^ School of Life Science, Shanxi University, Taiyuan, Shanxi, China; ^4^ Key Laboratory of Biodiversity Formation Mechanism and Comprehensive Utilization of the Qinghai-Tibet Plateau in Qinghai Province, School of Life Sciences, Qinghai Normal University, Xining, China; ^5^ Wolfson College, Oxford University, Oxford, United Kingdom

**Keywords:** gene evolution, genome structure, organelle assembly, phylogeny, population structure, gene order, genetic diversity, parasitic plants

This Research Topic focuses on studies of organelle genomes, which has been a highly active area of research during the last years. Our focus on organelle genomes fulfills their potential to perform full-scale comparisons for non-model species. This is because they are typically smaller than nuclear genomes, and modern sequencing techniques and bioinformatic approaches often allow researchers to reliably assemble and annotate them. Dozens of approaches have been published describing ways to perform assemblies and annotations of chloroplast and mitochondrial genomes, and how to create alignments for phylogenetic analyses. Comparisons of chloroplast genomes are especially important for lineages of plants with unusual lifestyles, such as parasitic plants.

The paper by Yu et al. describes a comparison of plastome genomes to detect genomic rearrangements and gene evolution in the Love Vines of the genus *Cassytha*. Love Vines comprise a distinct genus in the same family as Avocado (Lauraceae) and are considered an oddball in that group. This is because Love Vines have experienced radical morphological and lifestyle changes from the otherwise typical members of Lauraceae, that are characterized by being woody trees or shrubs. These radical changes include having a non-woody vine habit and for being hemiparasites, depending on acquiring water and some mineral nutrients from other plants. To explore the genomic changes involved in the chloroplast of these hemiparasitic vines, Yu et al. sequenced eight chloroplast genomes from two species of Love Vines (*Cassytha larsenii* and *C. filliformis*) and found that these parasites have smaller chloroplast genomes than the rest of the Lauraceae. These reductions are due to the loss of an inverted repeat segment, the loss of several *ndh* genes, and the presence of non-functional (pseudogenized) genes. All these genomic alterations from typical Lauraceae reflect the impact of a parasitic lifestyle, where species progressively become more dependent on the nutrients acquired from hosts, promoting the degeneration of housekeeping genes involved in nutrient production.

Zhang et al. sequenced the mitochondrial genome of the Asian freshwater fish *Osteochilus salsburyi*, which is a ray-finned carp or minnow (Cyprinidae: Labeoninae), a family characterized for lacking a stomach and teeth. *O. salsburyi* is found in parts of Laos, northern Viet Nam, and southern China, where it is locally consumed by people and therefore of economic importance. Given the lack of genomic resources available for the species, the authors sequenced its genome and conducted analyses to infer its phylogenetic position and optimal codon usage in its genome. To conduct the phylogenetic analyses, they used a multiple sequence alignment comparing all available mitochondrial genomes for the genus *Osteochilus* and living relatives and built a tree depicting the relationships between them. Moreover, it was possible to estimate the time of divergence between taxa included in the phylogeny, where *O. salsburyi* split from relatives at approximately 154 Mya.

The study of genomes can be also focused to individual genes, as shown in the paper by Yang et al., where authors investigated the evolution of the *CHS* gene in plants. The *CHS* gene is involved in the production of the chalcone synthase enzyme, which catalyzes the flavonoid biosynthetic pathway involved in plant growth and development, as well as in regulating response to environmental hazards. Despite its key importance for the proper development of plants, little is known on the phylogenetic position of the *CHS* gene across green plants. To perform this comparative study, Yang et al. identified members of the *CHS* family using BLAST to query amino acid sequences of the *CHS* gene from *Arabidopsis thaliana*, and by building a Hidden Markov Model profile of *CHS* domains used to search protein databases for homolog genes. Researchers found that the *CSH* gene originated before the rise of algae and diversified in species-rich lineages such as Poales, asterids, and Fabales. In spite of being present in virtually all lineages of green plants, the *CHS* gene has a highly conserved structure which reflects the importance of flavonoid biosynthesis in plants.

Beside analyzing whole organelles or individual genes, genomes can be also levered to examine population genetics as in the work presented by Li et al. Here, authors estimated the genetic diversity and population structure of two species of rats, the Brown Rat (*Rattus norvegicus*) and the Oriental Rat (*R. tanezumi*), using sequence data from microsatellites and one genomic region. Based on their analysis of short tandem repeats of nucleotides (i.e., microsatellites), the authors infer that Oriental Rats spreads slowly mainly in an unidirectionally way towards the north, whereas Black Rats spread faster and in multidirectional way. The speed of migration seems to be affected by habits unique to each species. While Oriental Rats prefer to nest above the ground, Brown Rats prefer ground level shelters. Also, authors argue that Brown Rats are apparently taking advantage of modern transportation infrastructure such as train rails and roads to expand their territory at a faster pace. The information generated in this study can help in monitoring migration routes for these two species of rats, given their importance as vectors for contagious diseases and as pests for causing damages to agriculture and infrastructures.

The collection of papers in this Research Topic provide readers with an overview of the potentials organelle genome analyses can offer to scientists. New sequencing technologies with long-read capacity is the next frontier, as it will allow more reliable and faster assemblies that will ease addition of new taxa and enrichment of genomic databases for broad-scale studies. Our Research Topic spans comparative analyses of genomes at various levels such as order and structure of genes across whole organelles, phylogenetic analyses of multiple organelles, evolution of a single gene across deep time scales, and inference of population genetic structures using short tandem repeats of nucleotides.

